# Dietary magnesium intake affects the association between dietary vitamin A and depression: a cross-sectional study

**DOI:** 10.3389/fnut.2025.1514681

**Published:** 2025-04-01

**Authors:** Canqun Yan, Conghui Gao, Kebin Zhan, Cheng Li

**Affiliations:** ^1^Health Management Center, The Second Affiliated Hospital, Hengyang Medical School, University of South China, Hengyang, China; ^2^Department of Neurology, The Second Affiliated Hospital, Hengyang Medical School, University of South China, Hengyang, China; ^3^Department of Emergency, The Affiliated Nanhua Hospital, Hengyang Medical School, University of South China, Hengyang, China

**Keywords:** magnesium intake, vitamin A, depression, cross-sectional study, National Health and Nutrition Examination Survey

## Abstract

**Introduction:**

Depression represents a significant global health burden, ranking as one of the leading causes of disability worldwide, and nutrition plays a key role in mental health. Vitamin A, essential for immune and neurological function, has shown conflicting associations with depression. Magnesium, essential for neurotransmission and neuroprotection, is associated with a reduced risk of depression. Importantly, magnesium is required for the activation of enzymes involved in vitamin A metabolism, suggesting a potential interaction between these nutrients in depression. However, this interaction remains poorly understood. This study investigates how magnesium intake modifies the relationship between vitamin A and depression, with the aim of elucidating their combined effects and informing personalized dietary strategies for depression prevention.

**Research design and methods:**

Data for this study were extracted from the National Health and Nutrition Examination Survey (NHANES) conducted between 2005 and 2016, involving a total of 60,936 participants. The final analysis included 25,277 adults aged ≥20 years (Female: 12,581, Male:12,696), excluding pregnant women, individuals under 20, and those with incomplete data. Depression was assessed using the Patient Health Questionnaire-9 (PHQ-9), supplemented by self-reporting questionnaires. Dietary intake was assessed via a recall interview at the mobile examination center (MEC). Dietary vitamin A intake, treated as a continuous variable, was categorized based on the median intake value. Stratified analyses were conducted based on sex and magnesium intake levels (Female: low: <310 mg/day; high: ≥310 mg/day; Male: low: <400 mg/day; high: ≥400 mg/day).

**Results:**

Our cross-sectional study showed that in women with low magnesium intake, higher vitamin A intake was associated with a reduced risk of depression (OR: 0.83, 95% CI: 0.76–0.92, *p* < 0.001), whereas no significant association was observed in the high magnesium group. Men did not show such an interaction. There was also a significant interaction between vitamin A levels and magnesium intake in reducing the incidence of depression (*p*-value for interaction = 0.145).

**Conclusion:**

Our study suggests that a sex-specific interaction between dietary magnesium and vitamin A in modulating depression risk. This interaction highlights the potential role of combined nutritional interventions in depression management. Further randomized controlled trials are warranted to confirm these findings.

## Introduction

Depression constitutes a major global public health challenge and is recognized as one of the leading contributors to disability-adjusted life years (DALYs) worldwide (1). According to the World Health Organization (WHO), approximately 280 million individuals globally are affected by depressive disorders, accounting for a substantial proportion of the global burden of disease (2). Depression is associated with significant morbidity, increased mortality rates, and profound impairments in health-related quality of life (HRQoL) (3). Its pervasive impact on both individual and societal levels underscores its status as a critical priority in mental health research and healthcare policy (4).

Concurrently, vitamin A deficiency has become increasingly prevalent, raising concerns about its broader health implications (5). Vitamin A deficiency, has significant health implications beyond its well-known roles in vision and immunity (5, 6). Emerging evidence suggests its involvement in neurological health, particularly in mood regulation and depression, mediated by oxidative stress and neuroinflammation—key pathways in depression pathogenesis (7, 8).

The relationship between vitamin A and depression is contentious. Recent studies have suggested a potential link between vitamin A and depression risk (7). Observational studies by Zhang indicated that vitamin A intake might reduce depression (9), while a study reported that excessive vitamin A intake could elevate depression risk and even suicidal ideation (10). The conflicting results of the two trials on vitamin A and depression may be due to important methodological differences: Study design: The former study was likely to have used observational methods to detect associations but not causality, whereas the latter may have used controlled designs such as RCTs to provide stronger causal evidence. Population: Differences in demographics or baseline health status may have influenced the results. Vitamin A assessment: The former study may have relied on self-reported dietary recall, which is prone to bias, while the latter may have used objective biomarkers such as serum retinol. Confounders: The former study may have inadequately controlled for confounding factors, whereas the latter is likely to have used more rigorous adjustments. Measurement of depression: The former study may have used self-report scales, while the latter may have used clinical diagnoses for greater accuracy. Dose–response: Variations in the range of vitamin A intakes studied (e.g., deficiency vs. excess) could lead to different results. These discrepancies may also arise from failing to account for confounding factors, such as dietary magnesium intake. These differences highlight the need for standardized methods to clarify the role of vitamin A in depression.

Magnesium is vital for cellular function, participating in numerous enzymatic reactions (11), intracellular signaling (12), myelination, synaptic formation and maintenance (13), and modulating neurotransmission of serotonin, norepinephrine, and dopamine. Research indicates that anxiety and depression are mediated by glutaminergic neurons in the hippocampus (14), and magnesium may regulate depression by influencing glutaminergic neurotransmission through its effects on N-methyl-D-aspartate (NMDA) receptors (15). Vitamin A, as a potent antioxidant, helps to mitigate oxidative damage, while magnesium stabilizes cell membranes and reduces excitotoxicity, which can exacerbate oxidative stress (13). Together they may synergistically protect against neuroinflammation and neuronal damage. In addition, magnesium is required for the activation of enzymes involved in vitamin A metabolism, suggesting that adequate magnesium levels are necessary for optimal vitamin A function (8). This interdependence means that magnesium deficiency could impair the neuroprotective effects of vitamin A, potentially increasing the risk of depression. Conversely, adequate magnesium intake may enhance vitamin A’s ability to modulate mood-related pathways (11). In conclusion, the interaction between magnesium and vitamin A in depression is biologically plausible, driven by their complementary roles in oxidative stress, neuroinflammation and neurotransmission. This synergy highlights the importance of considering both nutrients in depression prevention and treatment strategies. While studies have highlighted interactions between magnesium and vitamin A (8), clinical research on the impact of magnesium intake on the relationship between vitamin A and depression is scarce.

Our study is the first to examine how magnesium and vitamin A affect depression risk. Previous studies examined these nutrients separately, we show that their combined effects vary by magnesium status. Among females with low magnesium intake, higher vitamin A levels were inversely associated with depression, whereas this protective effect diminished in those with adequate magnesium intake. This suggests that magnesium modulates vitamin A’s neuroprotective effects. Our findings address a key research gap by highlighting nutrient interactions, which pave the way for personalized dietary strategies in depression prevention. More trials are needed.

## Methods

### Data sources and study population

Data for this study were derived from the National Health and Nutrition Examination Survey (NHANES). NHANES database, known for its large sample size, robust design, and stringent quality control, provides data on daily dietary intakes that are adequate for estimating and comparing average intakes across populations.^1^ Six NHANES cycles were used for this study: 2005–2006, 2007–2008, 2009–2010, 2011–2012, 2013–2014, and 2015–2016. A total of 60,936 participants were included in the study. Participants with unknown depression classification (*n* = 25,973), participants with dietary vitamin A, magnesium, and covariate deficiencies (*n* = 9,162), and participants who were pregnant and younger than 20 years (*n* = 418) were excluded. A total of 25,277 participants were finally included in the study. Of these, 12,581 were female patients and 12,696 were male patients. The study population comprised individuals over 20 years old who completed interviews and examinations at the mobile examination center (MEC). We excluded participants with incomplete data regarding dietary vitamin A levels, covariates, or depression status, direct deletion for values with less than 5% missing values. NHANES assesses the health and nutritional status of the non-institutionalized US population using a multistage, stratified probability sampling design to ensure a representative sample. Demographic and health history data were collected during household interviews, followed by more detailed interviews, physical examinations, and blood sample collections at the MEC. These samples were analyzed at the National Center for Environmental Health, Laboratory Sciences Division of the Centers for Disease Control and Prevention.

Ethical approval for the study was granted by the National Ethical Review Board for Health Statistics Research. The study protocol, which was approved by the Ethics Review Board (protocols #2005–06 and #2011–17), can be accessed on the NHANES Ethics Review Board website. Our research utilized publicly available NHANES data, with all details sourced from the official NHANES website.

### Measurement and classification of dietary intake

Dietary magnesium intake for the past 24 h was evaluated through a recall interview at the mobile examination center (MEC). Dietary vitamin A intake, considered a continuous variable, was classified into categories based on the median intake value. This classification approach was adopted in accordance with expert consensus reached during regular workshops focused on assessing NHANES data collection methods (16). The classification of magnesium intake is based on population-based dietary recommendations, the US Dietary Reference Intakes (DRIs), which suggest an Adequate Intake (AI) of 400 mg/day for adult men and 310 mg/day for adult women (17), This approach is consistent with previous studies examining the role of magnesium in health outcomes and ensures a balanced distribution for statistical analysis. The 24-h recall method, a widely accepted technique for dietary intake assessment in large-scale epidemiological studies, has been consistently utilized in NHANES due to its endorsement by expert panels during periodic reviews of survey methodologies (16). Comprehensive data on the collection and quantification of magnesium and vitamin A intake are available in the NHANES database.^2^

### Depression classification

The presence of depression was ascertained using the Patient Health Questionnaire-9 (PHQ-9) and supplementary self-report questionnaires (18). The PHQ-9 is a reliable self-report scale for depressive disorders, consistent with the nine DSM-IV diagnostic criteria established by the American Psychiatric Association. Participants were identified as having depression if their PHQ-9 scores ranged from 5 to 15, suggesting the presence of depressive symptoms; scores between 0 and 4 indicated the absence of depression (19).

### Covariates

Based on the literature and supported by clinical experience, the fully adjusted model included: age, gender, race, body mass index (BMI), activity, marital status, smoking status, alcohol consumption, hypertension, and diabetes. Race/ethnicity categories were non-Hispanic White, non-Hispanic Black, Mexican American, and other races. Marital status was categorized as married, cohabiting, or single. Educational attainment was classified into three levels: less than 9 years, 9–12 years, and more than 12 years of education. Household income was stratified into low, medium, and high groups based on the poverty income ratio (PIR) as defined by a US government report: low (PIR ≤ 1.3), medium (PIR > 1.3–3.5), and high (PIR > 3.5). Smoking status was categorized as never smokers (those who smoked fewer than 100 cigarettes in their lifetime), current smokers, and former smokers (those who quit after smoking more than 100 cigarettes). Physical activity levels were distinguished as non-vigorous and vigorous. Alcohol consumption was binary, defined as annual intake of at least 12 alcoholic beverages (yes) versus fewer than 12 (no), based on self-reported data from questionnaire interviews. BMI was calculated from height and weight measurements; weight was measured in pounds and converted to kilograms, while height was measured to the nearest millimeter using an electronic stadiometer. Diabetes and hypertension were identified based on specific criteria. Diabetes was diagnosed if any of the following conditions were met: self-reported diabetes, glycohemoglobin HbA1c >6.5%, fasting glucose ≥7.0 mmol/L, random blood glucose ≥11.1 mmol/L, 2-h oral glucose tolerance test (OGTT) blood glucose ≥11.1 mmol/L, use of diabetes medication or insulin, or a doctor’s diagnosis of type 1 diabetes at birth. Hypertension was defined according to the International Society of Hypertension standards and self-reported questionnaire data. Participants were classified as hypertensive if they were on hypertension medication, had a physician’s diagnosis, or had a real-time blood pressure measurement of ≥140/90 mmHg. Ambulatory blood pressure monitoring (ABPM) criteria for hypertension included a mean blood pressure of ≥130/80 mmHg over 24 h, daytime ≥135/85 mmHg, and nighttime ≥120/70 mmHg.

### Statistical analysis

The statistical analyses were conducted using R,^3^ Free Statistics software version 1.71 (20), and Empower Stats.^4^ For the stratified sampling data obtained from the NHANES database, we employed multiple logistic regression, stratified analysis, and sensitivity analysis as per the recommended statistical methods.^5^ The relationship between vitamin A levels and depression was explored using multivariate linear regression. Depression scores were evaluated across varying levels of magnesium intake. Interactions among subgroups were assessed using likelihood ratio tests, and 95% confidence intervals (CIs) were determined. The threshold for statistical significance was set at *α* = 0.05. Continuous variables were presented as mean ± standard deviation (SD) or median with interquartile range (IQR) for descriptive statistics, while categorical variables were expressed as weighted percentages (%). We utilized the chi-square test for categorical variables, t-test for normally distributed continuous variables, and Kruskal-Wallis test for continuous variables with skewed distributions.

## Results

### Baseline characteristics of the study population

**Figure 1:**
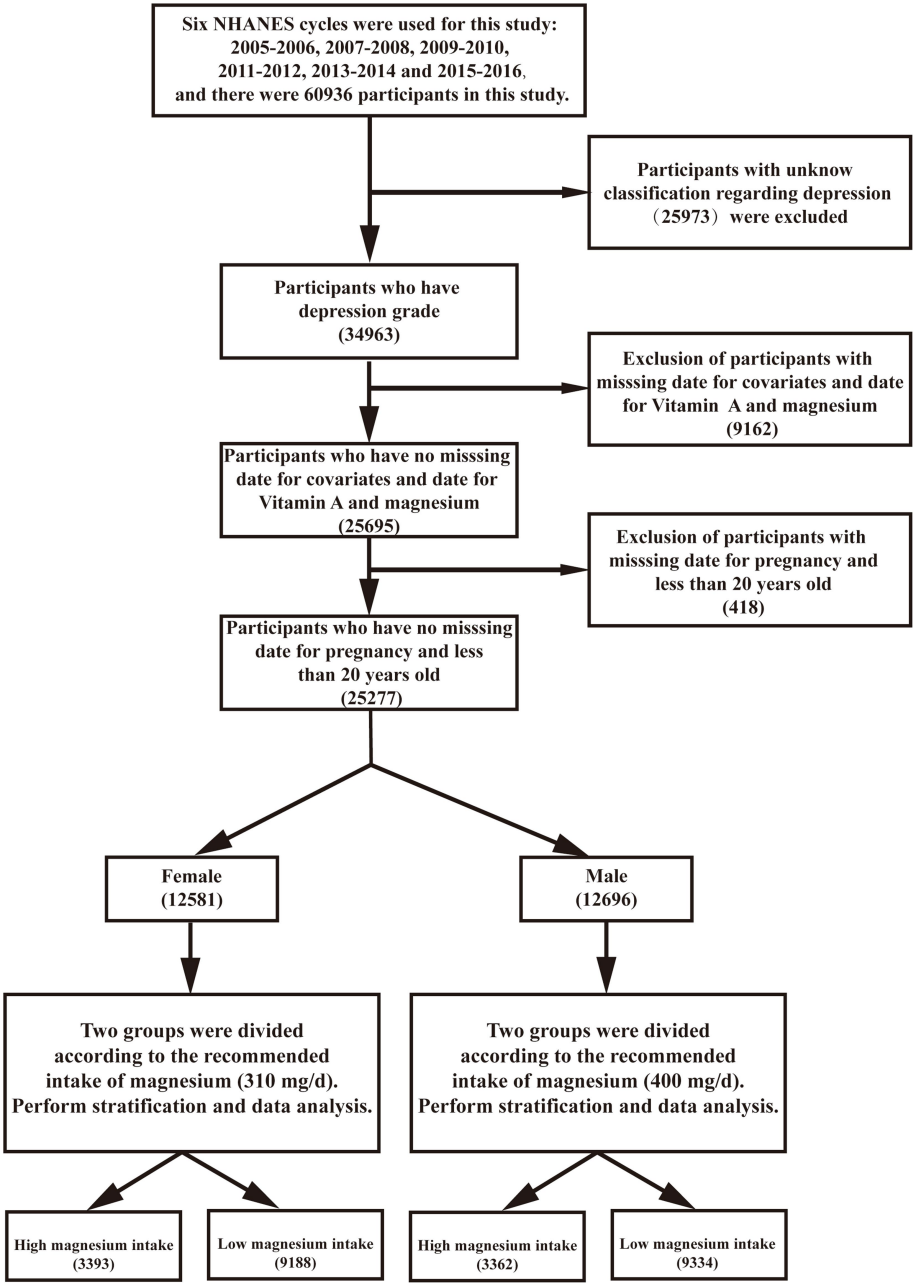
Experimental methods and procedures.

Six NHANES cycles 2005–2006, 2007–2008, 2009–2010, 2011–2012, 2013–2014, and 2015–2016 were used in this study, comprising an initial sample of 60,936 participants. 25,973 participants were excluded due to missing or incomplete depression status data, as assessed by the Patient Health Questionnaire-9 (PHQ-9). 9,162 participants were excluded due to incomplete data on dietary vitamin A, magnesium intake, or key covariates (e.g., age, gender, BMI, smoking status). 418 participants were excluded for being pregnant or under 20 years of age, as these factors could confound the analysis. After these exclusions, the final analytical sample consisted of 25,277 participants, whose data were used for all subsequent analyses. This sequential exclusion process is illustrated in Figure 1. Descriptive characteristics of participants according to their depression are shown in Table 1. Compared to non-depressed people, people with depression were more likely to be female, to have more education, to have a higher household income, to be married, to smoke less and to be less active. There was no significant difference in alcohol consumption and race (*p* > 0.05).

**Table 1:** Baseline characteristics of participants.

Covariates	Depressive state			
	Total (*n* = 25,277)	Non-depression (*n* = 19,122)	Depression (*n* = 6,155)	*p*-value
Gender, *n* (%)				<0.001
Male	12,696 (50.2)	10,198 (53.3)	2,498 (40.6)	
Female	12,581 (49.8)	8,924 (46.7)	3,657 (59.4)	
Age (year), Mean ± SD	49.4 ± 17.7	49.5 ± 17.9	48.9 ± 17.1	0.010
BMI, Mean ± SD	29.2 ± 6.9	28.8 ± 6.5	30.4 ± 8.0	<0.001
Race/ethnicity, *n* (%)				0.637
Non-Hispanic White	11,608 (45.9)	8,803 (46)	2,805 (45.6)	
Non-Hispanic Black	5,302 (21.0)	3,977 (20.8)	1,325 (21.5)	
Mexican American	3,846 (15.2)	2,924 (15.3)	922 (15.0)	
Others	4,521 (17.9)	3,418 (17.9)	1,103 (17.9)	
Education level (year), *n* (%)				<0.001
<9	2,452 (9.7)	1,742 (9.1)	710 (11.5)	
9–12	9,413 (37.2)	6,734 (35.2)	2,679 (43.5)	
>12	13,412 (53.1)	10,646 (55.7)	2,766 (44.9)	
Marital status, *n* (%)				<0.001
Married or living with a partner	15,121 (59.8)	12,005 (62.8)	3,116 (50.6)	
Living alone	10,156 (40.2)	7,117 (37.2)	3,039 (49.4)	
Family income, *n* (%)				<0.001
Low	7,868 (31.1)	5,176 (27.1)	2,692 (43.7)	
Medium	9,516 (37.6)	7,260 (38)	2,256 (36.7)	
High	7,893 (31.2)	6,686 (35)	1,207 (19.6)	
Drink, *n* (%)				0.468
No	6,978 (27.6)	5,301 (27.7)	1,677 (27.2)	
Yes	18,299 (72.4)	13,821 (72.3)	4,478 (72.8)	
Hypertension, *n* (%)				<0.001
No	17,784 (70.4)	13,883 (72.6)	3,901 (63.4)	
Yes	7,493 (29.6)	5,239 (27.4)	2,254 (36.6)	
Activity, *n* (%)				<0.001
Vigorous work activity	5,456 (21.6)	4,223 (22.1)	1,233 (20)	
Others	19,821 (78.4)	14,899 (77.9)	4,922 (80)	
DM, *n* (%)				<0.001
No	22,146 (87.6)	17,035 (89.1)	5,111 (83)	
Yes	3,131 (12.4)	2,087 (10.9)	1,044 (17)	
Smoking status, *n* (%)				<0.001
Never	13,569 (53.7)	10,796 (56.5)	2,773 (45.1)	
Current	6,314 (25.0)	4,857 (25.4)	1,457 (23.7)	
Former	5,394 (21.3)	3,469 (18.1)	1,925 (31.3)	
Vit A intake, Mean ± SD	605.9 ± 679.5	618.5 ± 695.3	566.7 ± 626.4	<0.001
Magnesium, Mean ± SD	295.2 ± 151.4	300.1 ± 150.9	280.0 ± 151.9	<0.001

### Magnesium intake modulates the association between dietary vitamin a and depression

Univariate analysis revealed that age, gender, BMI, education level, marital status, household income, physical activity, smoking, and dietary intakes of vitamin A and magnesium were significantly associated with depression (Table 2). Table 3 demonstrates the following: In Model 1, dietary vitamin A intake was significantly associated with depression risk, without adjustment for other variables (*p* < 0.001). When age and sex were adjusted for in Model 2, the association between dietary vitamin A intake and depression risk remained significant (*p* < 0.001). In Model 3, which included additional adjustments for race/ethnicity, BMI, physical activity, marital status, smoking status, alcohol consumption, hypertension, and diabetes, the association remained significant (*p* = 0.021). Furthermore, when magnesium intake was incorporated into Model 3, the results continued to exhibit a significant association (*p* = 0.145).

**Table 2:** Association of covariates and depression risk.

Covariate	OR (95% Cl)	*p*-value
Gender, *n* (%)		
Male	1 (reference)	
Female	1.67 (1.58–1.77)	<0.001
Age (year)	1 (1–1)	0.01
BMI (kg/m^2^)	1.03 (1.03–1.04)	<0.001
Race/ethnicity, *n* (%)		
Non-Hispanic White	1 (reference)	
Non-Hispanic Black	1.05 (0.97–1.13)	0.246
Mexican American	0.99 (0.91–1.08)	0.81
Others	1.01 (0.93–1.1)	0.757
Education level (year)		
<9	1 (reference)	
9–12	0.98 (0.88–1.08)	0.629
>12	0.64 (0.58–0.7)	<0.001
Marital status, *n* (%)		
Married or living with a partner	1 (reference)	
Living alone	1.65 (1.55–1.74)	<0.001
Family income, *n* (%)		
Low	1 (reference)	
Medium	0.6 (0.56–0.64)	<0.001
High	0.35 (0.32–0.38)	<0.001
Drink, *n* (%)		
No	1 (reference)	
Yes	1.02 (0.96–1.09)	0.468
hypertension, *n* (%)		
No	1 (reference)	
Yes	1.5311 (1.4408–1.6271)	<0.001
Activity, *n* (%)		
Vigorous work activity	1 (reference)	
Others	1.13 (1.05–1.21)	0.001
DM, *n* (%)		
No	1 (reference)	
Yes	1.67 (1.54–1.81)	<0.001
Smoking status, *n* (%)		
Never	1 (reference)	
Current	1.17 (1.09–1.25)	<0.001
Former	2.16 (2.02–2.32)	<0.001
Vit A intake	0.86 (0.82–0.91)	<0.001
Magnesium	0.91 (0.89–0.93)	<0.001

**Table 3:** The associations between vitamin A and depression risk.

	Total	Depression	OR_95CI	*p*-value
Model 1	25,277	6,155	0.86 (0.82–0.91)	<0.001
Model 2			0.89 (0.85–0.94)	<0.001
Model 3			0.94 (0.90–0.99)	0.021
Model 4			0.96 (0.91–1.01)	0.145

Model 1 unadjusted.

Model 2 adjusted for age, gender.

Model 3 adjusted for age, gender, race/ethnicity, BMI, activity, marital status, smoking status, alcohol, hypertension, DM.

Model 4 adjusted for age, gender, race/ethnicity, BMI, activity, marital status, smoking status, alcohol, hypertension, DM, magnesium.

After adjusting for age, gender, race/ethnicity, BMI, physical activity, marital status, smoking status, alcohol consumption, hypertension, and diabetes, magnesium intake demonstrated a significant interaction effect on the relationship between vitamin A and depression risk (Table 4). In females, a significant inverse association was observed between vitamin A intake and depression risk in the low magnesium intake group (≤310 mg/day) (OR: 0.9, 95% CI: 0.82–1, *p* = 0.047), whereas no significant association was found in the high magnesium intake group (>310 mg/day) (OR: 1.06, 95% CI: 0.95–1.18, *p* = 0.276). Subgroup analysis further revealed that in the low magnesium intake group, participants with higher vitamin A intake (>439 × 10^−3^ mg/day) had a significantly reduced risk of depression (OR: 0.83, 95% CI: 0.76–0.92, *p* < 0.001). In contrast, no significant association was observed in the high magnesium intake group (OR: 0.93, 95% CI: 0.78–1.12, *p* = 0.465).

**Table 4:** Effect of low and high magnesium intake groups on the association between vitamin A and depression.

Female					
Variable	Low magnesium intake <310 (mg/d) (*n* = 9,188)		High magnesium intake ≥310 (mg/d) (*n* = 3,393)		*p* for interaction
	OR (95% CI)	*p*-value	OR (95% CI)	*p*-value	
Vitamin A (mg)	0.9 (0.82–1)	0.047	1.06 (0.95–1.18)	0.276	0.032
Subgroups					
≤439 × 10^−3^ (mg)	1 (reference)		1 (reference)		
>439 × 10^−3^ (mg)	0.83 (0.76–0.92)	<0.001	0.93 (0.78–1.12)	0.465	

Male					
Variable	Low magnesium intake <400 (mg/d) (*n* = 9,344)		High magnesium intake ≥400 (mg/d) (*n* = 3,362)		*p* for interaction
	OR (95% CI)	*p*-value	OR (95% CI)	*p*-value	
Vitamin A (mg)	0.94 (0.85–1.03)	0.181	0.94 (0.84–1.06)	0.334	0.876
Subgroups					
≤496 × 10^−3^ (mg)	1 (reference)		1 (reference)		
>496 × 10^−3^ (mg)	0.92 (0.82–1.02)	0.115	0.91 (0.75–1.11)	0.342	

In males, no significant association was found between vitamin A intake and depression risk in either the low magnesium intake group (≤400 mg/day) (OR: 0.94, 95% CI: 0.85–1.03, *p* = 0.181) or the high magnesium intake group (>400 mg/day) (OR: 0.94, 95% CI: 0.84–1.06, *p* = 0.334). Similarly, subgroup analysis showed no significant differences in depression risk based on vitamin A intake levels in males (low magnesium group: OR: 0.92, 95% CI: 0.82–1.02, *p* = 0.115; high magnesium group: OR: 0.91, 95% CI: 0.75–1.11, *p* = 0.342).

## Discussion

The findings of this study highlight the significant role of magnesium intake in modulating the relationship between vitamin A and depression risk, particularly among females. In females with low magnesium intake, higher vitamin A intake was associated with a reduced risk of depression, whereas this association was not observed in those with high magnesium intake. This suggests that magnesium may play a critical role in mediating the antidepressant effects of vitamin A.

Our study found that in females with low magnesium intake (≤310 mg/day), higher vitamin A intake was associated with a reduced risk of depression (OR: 0.83, 95% CI: 0.76–0.92, *p* < 0.001). However, no significant association was observed in males. In previous Studies, a meta-analysis by Derom et al. reported that higher dietary magnesium intake was associated with a 22% reduction in depression risk (OR: 0.78, 95% CI: 0.69–0.88) (21). Another study by Tarleton et al. found that magnesium supplementation significantly improved depressive symptoms, with an Cohen’s d of 0.61 (95% CI: 0.32–0.90) (22). The effect size in our study is consistent with the protective effect of magnesium observed in previous research, though slightly smaller. This may be due to the focus on the interaction between magnesium and vitamin A, rather than magnesium alone.

A meta-analysis by Zhang et al. found that higher dietary vitamin A intake was associated with a 15% reduction in depression risk (OR: 0.85, 95% CI: 0.77–0.94) (9). Another study by Hu et al. suggested that vitamin A might exert antidepressant effects through its role in neuroplasticity and antioxidant activity, though specific effect sizes were not reported (7). The effect size in our study is comparable to the findings of Zhang et al. (9), suggesting that the protective effect of vitamin A on depression risk is consistent across studies. However, the interaction with magnesium intake adds a novel dimension to this relationship. The interaction between magnesium and vitamin A was significant in females (*p* for interaction = 0.032), with the protective effect of vitamin A being more pronounced in the low magnesium intake group. Limited research has directly examined the interaction between magnesium and vitamin A in relation to depression. However, studies have highlighted the synergistic role of these nutrients in neuroprotection and antioxidant activity. For example, Rabbani et al. found that co-supplementation with magnesium, zinc, and vitamin A improved oxidative stress markers and thyroid function, which are indirectly related to depression (8). The significant interaction observed in this study is novel and suggests that magnesium intake may modulate the antidepressant effects of vitamin A. This finding aligns with the broader literature on the synergistic role of micronutrients in mental health but highlights the need for further research to confirm these interactions.

This study adds to the growing body of evidence supporting the role of micronutrients in mental health and highlights the need for further research to explore the mechanisms underlying these interactions. Future studies should consider sex-specific differences and the potential for nutrient–nutrient interactions in the development of dietary guidelines for depression prevention and management.

The primary hypotheses regarding the etiology of depression include monoaminergic transmission disorders, increased glutaminergic excitotoxicity, neuroinflammation, oxidative stress, and neurotrophic factor deficiency. Oxidative stress is a significant factor in the pathogenesis of depression (23), and total dietary antioxidant capacity is negatively correlated with depression incidence. Both vitamin A and dietary magnesium possess significant antioxidant properties, suggesting a potential interaction between vitamin A and magnesium in the treatment of depression (24).

Emerging evidence suggests that vitamin A plays a critical role in brain health, particularly in modulating neuroplasticity, neurotransmitter systems, and oxidative stress pathways (7). However, its efficacy is context-dependent, influenced by factors such as magnesium intake, dosage, and sex. Dose–Response Complexity: While moderate vitamin A intake appears protective, excessive intake may increase depression risk. A U-shaped relationship has been proposed, emphasizing the need for precision in dietary recommendations (25). Sex Differences: Females may be more vulnerable to vitamin A dysregulation due to hormonal interactions (e.g., estrogen enhances retinol-binding protein synthesis) (26). This aligns with our sex-stratified findings, where vitamin A’s effects were significant only in females. Nutrient Synergy: Vitamin A rarely acts in isolation. Its effects are modulated by interactions with zinc, iron, and other antioxidants (27).

Magnesium is crucial for numerous cellular processes, including the regulation of numerous enzymatic reactions (28), intracellular signaling (29), myelination (30), synapse formation and maintenance (31), and neurotransmission involving serotonin, dopamine, and acetylcholine (32). Consequently, magnesium is implicated in the pathogenesis of depression. The interaction between magnesium intake and vitamin A’s association with depression can be attributed to biochemical synergy, neuroprotective pathway regulation, and sex-specific physiological factors. Vitamin A must be enzymatically converted to its active metabolite, retinoic acid, to exert neuroprotective effects. This process requires magnesium-dependent enzymes (13). Magnesium regulates NMDA receptors, low magnesium intake leads to NMDA hyperactivity, increasing neuronal damage and depressive symptoms (33). Vitamin A (via retinoic acid) enhances synaptic plasticity and neurogenesis in the hippocampus (34), potentially counteracting NMDA-driven excitotoxicity. This compensatory mechanism may explain why vitamin A’s protective effect is pronounced in low magnesium conditions. Estrogen enhances retinol-binding protein (RBP) synthesis, increasing retinol transport in females (35). This may amplify vitamin A’s bioavailability in women with low magnesium, magnifying its antidepressant effects. Females exhibit higher magnesium requirements due to menstrual cycles and hormonal fluctuations (36). Low magnesium intake may disproportionately affect women, creating a physiological state where vitamin A’s neuroprotective effects become more salient.

Our research has several limitations. Firstly, the cross-sectional design precludes the establishment of causality or directionality. Despite numerous adjustments, unmeasured variables may still confound the results. However, potential confounders, including dietary factors, were accounted for in the logistic regression model. Secondly, accurately measuring whole-body magnesium status is challenging. We relied on dietary interviews and 24-h recalls to assess magnesium intake, which are prone to recall bias due to their self-reported nature. Thirdly, while our study encompassed a large sample, it was limited to U.S. residents. Fourthly, while our study rigorously adjusted for major demographic and lifestyle confounders, residual confounding by unmeasured nutrients, dietary patterns, and socioeconomic stressors remains a limitation. For example, diets rich in vitamin A (e.g., liver, fish) often include omega-3 sources (e.g., fatty fish). The function of the anti-inflammatory and neuroprotective effects of omega-3 fatty acids may overlap with the magnesium/vitamin A pathways. Unadjusted omega-3 intake could inflate the apparent protective effect of vitamin A. The interaction between magnesium and vitamin A may reflect broader dietary or biochemical synergies rather than isolated effects. Future studies should adopt holistic dietary assessments and mechanistic validation to clarify these relationships. Finally, the select bias include exclusion criteria and missing Data, NHANES participation bias, sex and demographic representativeness, healthy participant bias is present in this study.

Therefore, caution is advised when generalizing these findings to other populations. Recommendations for Future Studies: 1. Prospective Cohorts: Longitudinal designs with repeated dietary assessments can clarify temporal relationships and reduce reverse causation bias. 2. Biomarker Validation: Combining dietary recalls with biomarkers (e.g., serum retinol, erythrocyte magnesium) can improve nutrient status assessment. 3. Sensitivity Analyses: Conduct analyses using multiple imputation or inverse probability weighting to evaluate the impact of missing data. 4. Stratified Sampling: Oversample high-risk populations (e.g., low-income, institutionalized individuals) to enhance representativeness. Future research should prioritize randomized controlled trials to clarify causal relationships and optimize therapeutic thresholds for vitamin A in depression management.

The results of our study support a paradigm shift toward nutrient interaction-aware clinical practices in depression management. By incorporating magnesium and vitamin A status into risk assessment and therapeutic algorithms, clinicians can advance precision nutrition strategies. However, real-world implementation requires rigorous RCT validation, cost–benefit analysis, and interdisciplinary collaboration between nutritionists, psychiatrists, and public health experts.

## Conclusion

Our study demonstrates that magnesium intake modifies the relationship between dietary vitamin A and depression risk in a sex-specific manner. Among females with low magnesium intake, higher vitamin A levels were inversely associated with depression, whereas this protective effect diminished in those with adequate magnesium intake. No significant associations were observed in males. The interaction underscores the synergistic roles of magnesium and vitamin A in neuroprotection, potentially mediated through oxidative stress reduction and NMDA receptor regulation. These findings emphasize the need for personalized dietary recommendations that consider nutrient interactions, particularly in populations at risk of magnesium deficiency. Future research should prioritize longitudinal designs, biomarker validation, and clinical trials to establish causality and refine therapeutic thresholds for micronutrient interventions in depression management.

## Data Availability

The original contributions presented in the study are included in the article/supplementary material, further inquiries can be directed to the corresponding authors.

## Footnotes

^1^ https://dietassessmentprimer.cancer.gov/profiles/recall

^2^ https://wwwn.cdc.gov/Nchs/Nhanes/2005-2006/DR1IFF_D.htm

^3^http://www.R-project.org, The R Foundation.

^4^ http://www.empowerstats.com

^5^ https://wwwn.cdc.gov/nchs/nhanes/AnalyticGuidelines.aspx

## References

[1] Moreno-Agostino D Wu Y-T Daskalopoulou C Hasan MT Huisman M Prina M. Global trends in the prevalence and incidence of depression: a systematic review and meta-analysis. J Affect Disord. (2021) 281:235–43. doi: 10.1016/j.jad.2020.12.035 33338841

[2] Depressive disorder (depression). Available online at: https://www.who.int/news-room/fact-sheets/detail/depression (Accessed February 28, 2025).

[3] IsHak WW Mirocha J James D Tobia G Vilhauer J Fakhry H. Quality of life in major depressive disorder before/after multiple steps of treatment and one-year follow-up. Acta Psychiatr Scand. (2015) 131:51–60. doi: 10.1111/acps.12301, PMID: 24954156 PMC4267902

[4] Sartor C. Mental health and lived experience: the value of lived experience expertise in global mental health. Camb Prisms Glob Ment Health. (2023) 10:e38. doi: 10.1017/gmh.2023.24, PMID: 37854403 PMC10579645

[5] Zhao T Liu S Zhang R Zhao Z Yu H Pu L. Global burden of vitamin a deficiency in 204 countries and territories from 1990-2019. Nutrients. (2022) 14:950. doi: 10.3390/nu14050950, PMID: 35267925 PMC8912822

[6] Amimo JO Michael H Chepngeno J Raev SA Saif LJ Vlasova AN. Immune impairment associated with vitamin a deficiency: insights from clinical studies and animal model research. Nutrients. (2022) 14:5038. doi: 10.3390/nu14235038, PMID: 36501067 PMC9738822

[7] Hu P van Dam A-M Wang Y Lucassen PJ Zhou J-N. Retinoic acid and depressive disorders: evidence and possible neurobiological mechanisms. Neurosci Biobehav Rev. (2020) 112:376–91. doi: 10.1016/j.neubiorev.2020.02.013, PMID: 32070693

[8] Rabbani E Golgiri F Janani L Moradi N Fallah S Abiri B. Randomized study of the effects of zinc, vitamin a, and magnesium co-supplementation on thyroid function, oxidative stress, and hs-CRP in patients with hypothyroidism. Biol Trace Elem Res. (2021) 199:4074–83. doi: 10.1007/s12011-020-02548-3, PMID: 33409923

[9] Zhang Y Ding J Liang J. Associations of dietary vitamin a and beta-carotene intake with depression. A meta-analysis of observational studies. Front Nutr. (2022) 9:881139. doi: 10.3389/fnut.2022.881139, PMID: 35548582 PMC9083456

[10] Zhang L Xu Y Li X Yang F Wang C Yu C. Multivitamin consumption and childhood asthma: a cross-sectional study of the NHANES database. BMC Pediatr. (2024) 24:84. doi: 10.1186/s12887-024-04540-5, PMID: 38297283 PMC10829257

[11] Patel V Akimbekov NS Grant WB Dean C Fang X Razzaque MS. Neuroprotective effects of magnesium: implications for neuroinflammation and cognitive decline. Front Endocrinol. (2024) 15:1406455. doi: 10.3389/fendo.2024.1406455, PMID: 39387051 PMC11461281

[12] Stangherlin A O’Neill JS. Signal transduction: magnesium manifests as a second messenger. Curr Biol CB. (2018) 28:R1403–5. doi: 10.1016/j.cub.2018.11.003, PMID: 30562536

[13] de Baaij JHF Hoenderop JGJ Bindels RJM. Magnesium in man: implications for health and disease. Physiol Rev. (2015) 95:1–46. doi: 10.1152/physrev.00012.2014, PMID: 25540137

[14] Riaza Bermudo-Soriano C Perez-Rodriguez MM Vaquero-Lorenzo C Baca-Garcia E. New perspectives in glutamate and anxiety. Pharmacol Biochem Behav. (2012) 100:752–74. doi: 10.1016/j.pbb.2011.04.010, PMID: 21569789

[15] Pochwat B Szewczyk B Sowa-Kucma M Siwek A Doboszewska U Piekoszewski W. Antidepressant-like activity of magnesium in the chronic mild stress model in rats: alterations in the NMDA receptor subunits. Int J Neuropsychopharmacol. (2014) 17:393–405. doi: 10.1017/S1461145713001089, PMID: 24067405

[16] Ahluwalia N Dwyer J Terry A Moshfegh A Johnson C. Update on NHANES dietary data: focus on collection, release, analytical considerations, and uses to inform public policy. Adv Nutr Bethesda Md. (2016) 7:121–34. doi: 10.3945/an.115.009258, PMID: 26773020 PMC4717880

[17] Institute of Medicine (US) Standing Committee on the Scientific Evaluation of Dietary Reference Intakes. Dietary reference intakes for calcium, phosphorus, magnesium, vitamin D, and fluoride. Washington (DC): National Academies Press (US) (1997). 23115811

[18] Levis B Sun Y He C Wu Y Krishnan A Bhandari PM. Accuracy of the PHQ-2 alone and in combination with the PHQ-9 for screening to detect major depression: Systematic review and meta-analysis. JAMA. (2020) 323:2290–2300. doi: 10.1001/jama.2020.6504 32515813 PMC7284301

[19] Tarleton EK Littenberg B. Magnesium intake and depression in adults. J Am Board Fam Med Jabfm. (2015) 28:249–56. doi: 10.3122/jabfm.2015.02.140176, PMID: 25748766

[20] Yang Q Zheng J Chen W Chen X Wen D Chen W. Association between preadmission metformin use and outcomes in intensive care unit patients with sepsis and type 2 diabetes: a cohort study. Front Med. (2021) 8:640785. doi: 10.3389/fmed.2021.640785, PMID: 33855034 PMC8039324

[21] Derom M-L Sayón-Orea C Martínez-Ortega JM Martínez-González MA. Magnesium and depression: a systematic review. Nutr Neurosci. (2013) 16:191–206. doi: 10.1179/1476830512Y.0000000044 23321048

[22] Tarleton EK Littenberg B MacLean CD Kennedy AG Daley C. Role of magnesium supplementation in the treatment of depression: a randomized clinical trial. PLoS One. (2017) 12:e0180067. doi: 10.1371/journal.pone.0180067, PMID: 28654669 PMC5487054

[23] Salim S. Oxidative stress and psychological disorders. Curr Neuropharmacol. (2014) 12:140–7. doi: 10.2174/1570159X11666131120230309, PMID: 24669208 PMC3964745

[24] Milajerdi A Keshteli AH Afshar H Esmaillzadeh A Adibi P. Dietary total antioxidant capacity in relation to depression and anxiety in Iranian adults. Nutr Burbank Los Angel Cty Calif. (2019) 65:85–90. doi: 10.1016/j.nut.2018.11.017, PMID: 31077947

[25] Prakash R. The acute and chronic toxic effects of vitamin a. Am J Clin Nutr. (2006) 84:462; author reply 462–463. doi: 10.1093/ajcn/84.2.462, PMID: 16895899

[26] Olson JA. Serum levels of vitamin a and carotenoids as reflectors of nutritional status – PubMed. J Natl Cancer Inst. (1984) 73:1439–1444. 6439934

[27] Palmer AC Bedsaul-Fryer JR Stephensen CB. Interactions of nutrition and infection: the role of micronutrient deficiencies in the immune response to pathogens and implications for child health. Annu Rev Nutr. (2024) 44:99–124. doi: 10.1146/annurev-nutr-062122-014910, PMID: 38724105

[28] Cowan JA. Structural and catalytic chemistry of magnesium-dependent enzymes. Biometals Int J Role Met Ions Biol Biochem Med. (2002) 15:225–35. doi: 10.1023/a:1016022730880 12206389

[29] Pergolini I Schorn S Friess H Demir IE. The role of magnesium in acute pancreatitis and pancreatic injury: a systematic review. Visc Med. (2024) 40:264–75. doi: 10.1159/000540507, PMID: 39398394 PMC11466448

[30] Seyama T Kamei Y Iriyama T Imada S Ichinose M Toshimitsu M. Pretreatment with magnesium sulfate attenuates white matter damage by preventing cell death of developing oligodendrocytes. J Obstet Gynaecol Res. (2018) 44:601–7. doi: 10.1111/jog.13568, PMID: 29363221

[31] Yamanaka R Shindo Y Oka K. Magnesium is a key player in neuronal maturation and neuropathology. Int J Mol Sci. (2019) 20:3439. doi: 10.3390/ijms20143439, PMID: 31336935 PMC6678825

[32] Spasov AA Iezhitsa IN Kravchenko MS Kharitonova MV. Features of central neurotransmission in animals in conditions of dietary magnesium deficiency and after its correction. Neurosci Behav Physiol. (2009) 39:645–53. doi: 10.1007/s11055-009-9182-y, PMID: 19621270

[33] Murck H. Ketamine, magnesium and major depression--from pharmacology to pathophysiology and back. J Psychiatr Res. (2013) 47:955–65. doi: 10.1016/j.jpsychires.2013.02.015 23541145

[34] McCaffery P Zhang J Crandall JE. Retinoic acid signaling and function in the adult hippocampus. J Neurobiol. (2006) 66:780–91. doi: 10.1002/neu.20237, PMID: 16688774

[35] Elfimova AE Tipisova EV Bichkaeva FA Alikina VA Vlasova OS Gretskaya TB. Analysis of vitamin a and thyroid blood levels in the indigenous and local caucasian population of the arctic. Vopr Pitan. (2024) 93:14–24. doi: 10.33029/0042-8833-2024-93-5-14-24, PMID: 39563145

[36] Gao X Sun Y Huang X Zhou Y Zhu H Li Q. Adequate dietary magnesium intake may protect females but not males older than 55 years from cognitive impairment. Nutr Neurosci. (2024) 27:184–95. doi: 10.1080/1028415X.2023.2169986, PMID: 36803323

